# Supplementary material for: Bionanocellulose/Poly(Vinyl Alcohol) Composites Produced by In-Situ Method and Ex-Situ/Impregnation or Sterilization Methods
Source: Materials (Basel). 2021 Oct 23;14(21):6340. doi: 10.3390/ma14216340 (PMC8585208; doi:10.3390/ma14216340)
Supplement: Supplementary file 1 [file materials-14-06340-s001.zip › materials-1425939-supplementary.pdf]

materials

Article

Bionanocellulose/Poly(Vinyl Alcohol) Composites Produced by *In-Situ* Method and *Ex-Situ*/Impregnation or Sterilization Methods

Aldona Długa [1], Jolanta Kowalonek [2,*] and Halina Kaczmarek [2]

[1] Bowil Biotech Sp. z.o.o., 7 Skandynawska St., 84-120 Władysławowo, Poland; dluga_aldona@wpl.pl
[2] Faculty of Chemistry, Nicolaus Copernicus University in Toruń, 7 Gagarina St., 87-100 Toruń, Poland; halina@umk.pl
* Correspondence: jolak@umk.pl; Tel.: +48-56-6114-552

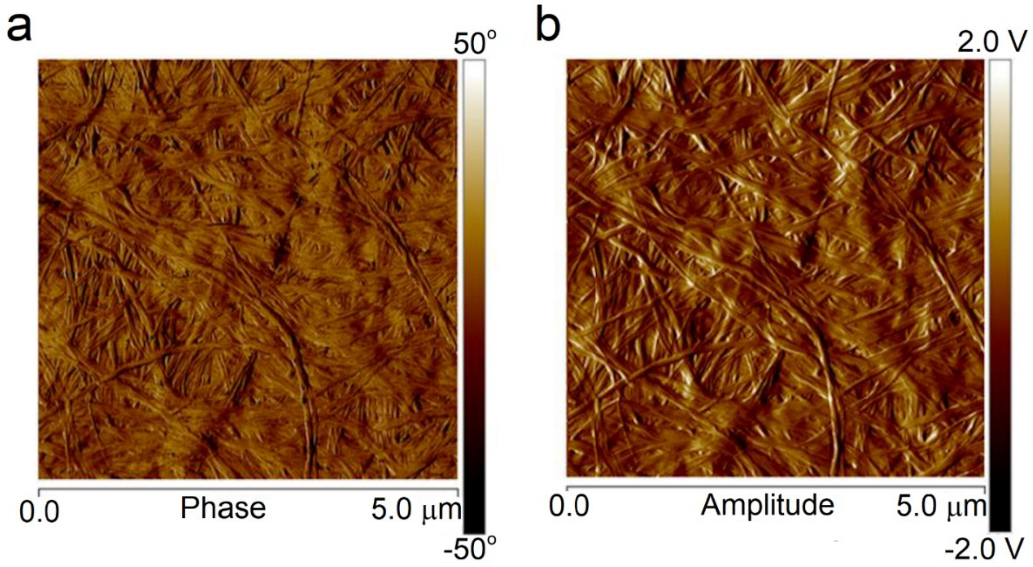

Figure S1. 2D-AFM images of BNC: phase (**a**), amplitude (**b**).

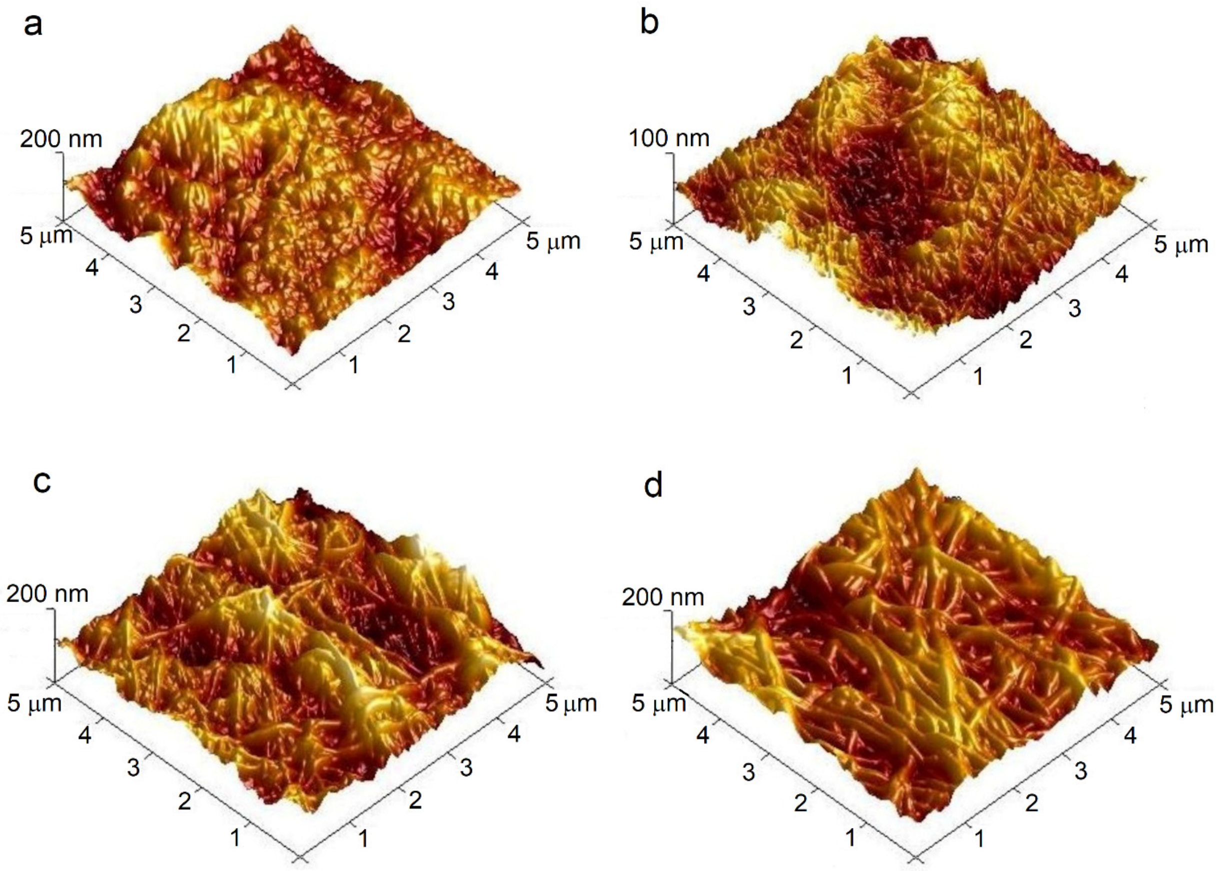

Figure S2. 3D-AFM images of BNC (**a**) and BNC/PVA composites obtained by *in-situ* (**b**), *ex-situ*/impregnation (**c**) and *ex-situ*/sterilization (**d**) method (at 4% PVA).
